# Editorial and Miscellaneous

**Published:** 1855-10

**Authors:** 


### EDITORIAL AND MISCELLANEOUS.

### OUR JOURNAL.

While we cannot by any means boast of a large subscription list, nor as yet a large corps of contributors, we do feel encouraged by the manifestations of interest received from various quarters. That we shall soon have a sustaining list of subscribers, and an abundant supply of original matter, we do not doubt. The circumstances under which we have been placed enable us to say that we can do something better so soon as we get into full operation. The first number was gotten up in a very hasty manner, and was necessarily very imperfect in many particulars, and while we have had more time to devote to the present, we are not enjoying the benefit of exchanges to any extent, and must, therefore, still claim the indulgence of our friends.

The present number of this Journal will only be sent to regular subscribers, or to those that we expect to take it. Any individual receiving it, and who is unwilling to be placed on our list, will please return the number, or, if he prefers it, notify us of the fact by letter.

We again repeat that our object is to establish a free medical press, and renew our solicitations to all regular and true medical men to become contributors.

Independently of the benefit to ourselves from a free and full interchange of opinions and experience, there is no quarter of the world more interesting than our own in connection with medical sciences, and there is in lavish profusion medical talent and skill in the South equal to that to be found anywhere.

There is nothing wanting to make us perfectly independent in every particular but the energy to use the advantages we have in our possession.

We have no fault to find, or quarrel to make, so far at least as medicine is concerned, with the northern portion of our confederacy, for though they have been built up to a great extent at our own expense, the fault is our own; in this matter their only offence has been in simply receiving the patronage so liberally *forced* upon them, and by which they have had the inducements offered, and the means furnished, for supplying advantages which were not presented here, because our people have preferred building up others rather than themselves.

When we take a view of the vast extent, the undeveloped and multiplied resources of the State of Georgia alone, constituting an empire within itself, we feel that there is a sufficient field within our own borders to sustain in the most successful manner our whole enterprise; but calculating as we do most confidently upon the whole Southern country for encouragement and patronage, believing that we shall be able to return an equivalent, we could not well have a more magnificent prospect than that which opens before us, in connection with the medical institution of which this journal is the representative.

---

### YELLOW FEVER.

While we do not feel prepared to present any very matured views in reference to the etiology of Yellow fever, or to decide even for ourselves positively between the opposite doctrines which have prevailed upon this subject, whether it be true that it has been invariably imported into Europe and this country, whether it is absolutely contagious under all circumstances, propagated by fomites or a specific morbid emanation from the bodies of the sick, under certain conditions of impurity and elevation of temperature in the atmosphere of particular localities, or upon the other hand that it is non-contagious in its character, and that it is of local origin, still we cannot forbear to direct attention to the facts connected with the introduction of the disease which has been so destructive at Norfolk and Portsmouth.

While of course it is very difficult to obtain the precise facts connected with an epidemic during the confusion attending its continuance, the statements which follow emanate from one of the most prominent and reliable medical journals of the country,* and one published near the afflicted cities.

* Virginia Medical and Surgical Journal.

It seems that on the 7th of June the steamer "Ben. Franklin" arrived at the harbor of Hampton Roads, in the vicinity of Portsmouth. It being known to the authorities that the steamer had come from the Island of St. Thomas, where Yellow fever was then prevailing, careful inquiries were made as to the health of those on board. Ascertaining from the captain that they had no fever on the vessel, they imposed a quarantine of only twelve days, at the expiration of which time, on the twentieth of the month, the vessel was allowed to come up to the ship yard, adjoining the Government Navy Yard at Gosport, convenient to the city of Portsmouth.

On the fifth of July (twenty-eight days from the arrival of the vessel at the roads) the captain, (it is said in express violation of orders,) proceeded to unload his vessel, and in less than two days afterwards one of the crew was taken with Yellow fever. The next day six more were added to the list, and in a short time the Government Navy Yard was invaded by the destroyer, thus apparently released from the vessel. Here the work of death was so rapid and alarming, that terror seized the operatives and they fled in all directions. From the Navy Yard the fever soon extended to the neighboring city of Portsmouth, and "on the twenty-fifth day of July a resident of the infected district found his way to Norfolk, and the violent poison, taking fresh root midst the filthy purlieus and crowded population of 'Barry's row,' now reigned triumphantly on both banks of the river."

Here the disease continued to spread until about the first of September it was said that probably not less than a thousand persons were laboring under the fever in the two cities. If these are facts, (and they come well authenticated,) it would be difficult to make a stronger argument in favor of the contagiousness of this awful pestilence, than is presented in this brief detail.

We are taking steps to ascertain beyond doubt the whole truth connected with this matter, which will be laid before our readers at the earliest practicable day. At present, we are strongly impressed with the idea that the fever was introduced from the Island of St. Thomas, and that whatever favorable circumstances for its spread may have existed in the locality alluded to, it is not at all probable that there would have been Yellow fever developed but for the visit of the infected vessel.

We cannot, therefore, number ourselves among those who deprecate a return to the "expense, discomfort and folly of a system of rigid quarantine," preferring this, as we do, to incurring the slightest risk of the invasion of this devastating pestilence.

That certain conditions connected with temperature and an atmosphere loaded with malaria and the exhalations arising from our horribly filthy towns, should greatly favor the introduction, spread, and fatality of Yellow fever, we do not doubt; and while we would urge in the strongest possible manner the great importance of ventilation, sewerage, &c., we are the decided advocates of quarantine restrictions; indeed, it seems to us that the people who are exposed, and neglect this precaution, are guilty of the greatest folly.

---

### DRUGGISTS, PURE MEDICINES, &c.

We take great pleasure in calling the attention of the profession to an article of Quinine, the manufacture of C. ZIMMER, in Frankfort-on-the-Maine, a specimen of which has been laid upon our table by Mr. J. M. RANTIN, Druggist, Whitehall street, Atlanta, Ga., who keeps it on hand regularly for sale.

This article we have both inspected and used, and have no hesitation in recommending it as uncommonly pure and free from the many adulterations which are practised to so large an extent in this important and costly remedy. We hope that those druggists who are manifesting a laudable desire to furnish pure medicines, will meet with the encouragement which can alone sustain them in this honorable effort. To such an extent are we imposed upon in our medicinal agents at the present day, that the physician who does not direct particular attention to the character of the drugs used in his practice, will most certainly be subjected constantly to disappointment in their administration; and while upon this subject we would take the liberty of urging upon the physicians of the city and the surrounding country, the importance of sustaining the druggists here. In every point of view, it is best for the good of all to build up home interests.

And again it may be stated as an invariable rule everywhere, that the character of the medicinal agent furnished by the druggist is regulated by the demand of the physician—and for this highly culpable state of things, it cannot be disputed, that the latter is equally responsible with the former. If he is satisfied with, or prefers an inferior article, either from carelessness upon his part or because it is obtained at an inferior price, it is not to be disguised that he will be accommodated, whether he order his supply from New York, Philadelphia, or elsewhere.

It is only necessary, therefore, to be prepared to judge of the quality of an article and to *require* the best, and be willing to remunerate your druggist, and it will be furnished as well in Atlanta as in London or Paris; and from some experience and observation, we have no hesitation in asserting that there is no more difficulty in obtaining reliable medicines here, than at any other point.

At the same time, it is also true that our druggists present a slender claim to the support of the medical profession, dealing as they do so extensively in the patent nostrums of the day, and in many instances usurping the business of the physician in prescribing for disease.

It is hoped that some one will yet be found in Atlanta who will forego the pecuniary profit attending this morally contraband trade, so entirely antagonistic to the interests of true medicine—whether considered with reference to prescribing or compounding—and that the profession will, in a body, sustain such a course by their patronage.

---

### MANUAL OF CLINICAL MEDICINE, &c.

We have received from the publishers, Messrs. Blanchard & Lea, of Philadelphia, a small work entitled "A Manual of Clinical Medicine and Physical Diagnosis," by T. H. Tanner, M. D., Licentiate of the Royal College of Physicians, Physician to the Hospital for Women, &c., &c., to which is added the Code of Ethics of the American Medical Association. We have recently received and noticed a work of somewhat similar character by Thos. S. Powell, M. D., of Sparta, Ga., and had really supposed that there was no farther demand for compends of medicine, but we are compelled to admit, upon an examination of this work, that we have here again something worthy of the particular attention of the student of medicine as well as the practitioner.

---

EXCHANGES RECEIVED.—Boston Medical and Surgical Journal, Memphis Medical Recorder, New York Medical Times, Southern Journal of Medical and Physical Sciences.

---

*Prof. Austin Flint, M. D.*—We have been favored with an engraved likeness of the above named gentleman which was gotten up by his professional friends in Buffalo in testimony of their respect for him as a man, for his talents as an editor, and his acquirements as a physician. He has an appointment to one of the chairs in the Institution at Louisville, which he has accepted, and to which place he will soon remove. Dr. Hunt occupies the position in relation to the Medical Journal once so eminently filled by Dr. Flint; and, although too much cannot be said in behalf of Dr. Flint as an editor, still we are assured the Journal will not lose a tithe of the interest for which it has been heretofore so fully characterized.—*Iowa Medical Journal.*

*Iodine in Fibrous Tumors of the Uterus.*—We observe that Dr. West almost invariably orders for those of his patients at St. Bartholomew's, who are the subjects of fibrous tumors of the uterus, a long course of one or other of the preparations of iodine. The following is the prescription which was ordered for a middle-aged woman, who applied with that disease on Saturday: Potassii iodidi, gr. j.; syrupi ferri iodidi, m. xx.; aquæ carui, ℥ ss. Ter die sumend.

Dr. West remarked at the time that were the patient one in the higher ranks of life she would be just the one likely to be benefited by being sent to drink Kreuznach waters, which contain iodides and also bromides. In common with Dr. Rigby and other physicians, Dr. West entertains a high opinion of the value of the iodides in procuring the diminution of these tumors.—*London Med. Times and Gazette.*

*A New Method of Lithotomy in Women.*—By M. Vallet, Surgeon to the Hotel Dieu at Orleans. The peculiarity of this operation is, that the incision through the vesico-vaginal septum is made *transversely*, and that the incision is closed by suture immediately after the extraction of the stone. In making the incision, M. Vallet avails himself of the help afforded by a sound, of which half the thickness of the lower fifth is made to turn upon a central axis, so as to form a cross by being arranged transversely to the longitudinal axis of the instrument. This sound is opened after it is introduced into the bladder, and then, on making a little traction, the cross-piece is brought transversely across the outlet of the bladder, and thus a transverse projection is made in the vagina in the position in which the incision has to be made. This cross-piece, indeed, is the guide for the knife, and it is grooved for the purpose.

The ease with which it may be performed, and the diminished chance of urinary fistula, are said to be the advantages of this operation. It certainly answered well in two cases, which are related by the author in his present paper. *Nashville Med. and Surg. Jour. Gaz. Hebdom.*

*Tape Worm Expelled by Infusion of Pumpkin Seed.*—By D. Leasure, M. D., of New Castle, Pa.—In this Journal for July, 1854, I reported a case of tænia expelled by infusion of pumpkin seeds, and mentioned incidentally that the patient's mother had, at an early period of her life, passed a tape worm under the use of a "secret vegetable remedy, probably male fern."

On the 6th day of April last I was somewhat surprised to receive from the old lady a tape worm twenty-one feet eight inches long, in every respect similar to the one passed by her daughter a little less than a year previously. The head in this instance also was attached to the worm, showing its entire expulsion.

She informed me that, having discovered joints of the worm in her dejections, she resorted to the infusion of the pumpkin seeds on her own responsibility, and took it in the same manner as previously taken on my prescription by her daughter, viz: a pint of the bruised seed to be infused in three pints of boiling water, and left over night, the whole to be taken next day, the patient fasting in the meantime.—*Am. Jour. Med. Sci.*

*M. Valleix.*—This distinguished physician died of malignant sore throat on the 12th of July last, after a short illness. His remains were accompanied to the cemetery of Mont Parnasse by a large number of devoted friends. The orations at the tomb were delivered by MM. Barth, Goupil, Latour, and his old master, Louis, who was much affected. M. Valleix was a man of great erudition, and intimately acquainted with the contemporary medical literature of England. To strangers he was accessible and polite, and to not a few in this our northern metropolis, his memory is endeared by many acts of friendship. His chief works are—the *Guide du Medicin Practicien*, the *Traite des Neuralgies*, and the *Clinique des Maladies des Enfants nouveau-nes.*—*Edinburgh Med. Jour.*

*Twins Born at an Interval of Forty Days.*—(Med Neuigkeiten und Ann. der Medizin.) A country woman, 34 years of age, of good health, usually regular in her menstrual periods, primipara, gave birth, after an easy and regular labor, to a child, which, although completely developed, was weakly. It died eight days afterwards from the effects of cold. The placenta came away naturally, an hour after the birth of the child. A few hours subsequent, the woman attended to her domestic concerns. The swelling of the abdomen had only partially subsided; soon after, active movements continued to be perceived by the mother; there was neither secretion of milk, nor lochia, and no fever. Nothing of particular moment occurred in the case of this woman, until the expiration of an interval of forty days, at which period she gave birth to a second child, which, although feeble, had evidently arrived at the full term. At this period, and for the first time, the lochial flux and milk secretion made their appearance.—*N. O. Med. News and Hosp. Gaz.*

*Hydrocele.*—Prof. Langenbeck, of Berlin, not being satisfied with the effects of the iodine tincture as an injection in hydrocele, has recently been employing chloroform as a substitute with the happiest results. He finds that it produces adhesive inflammation more quickly and more surely than the old remedy. After withdrawing the fluid of hydrocele, he injects about one drachm of chloroform, which remains for a short time, and then is allowed to escape. Langenbeck, in the Deutshe Klinik, reports four cases treated thus, which were radically cured in two or three weeks.—*Western Lancet.*

*Extraction of a Uterine Pessary from the Bladder.*—By Dr. Uytterhoeven.—This case occurred in a young woman who, on account of uterine displacement, required the use of a pessary *a tige.* Some time having elapsed after the introduction of the instrument, during which period the patient had neglected to withdraw and clean it, ulceration took place, and gradually perforated the vesico-vaginal wall. The pessary thus opened a passage for itself, by which its ivory head became introduced within the bladder, whilst the stem, which was of metal, was tightly grasped by the fistulous opening, and remained in the vagina.

The attempts to break the "cuvette" or head of the instrument having failed, M. Uytterhoeven, after placing the patient under the influence of chloroform, slipped a curved probe-pointed bistoury along its stem, and, guided by the index finger, entered it within the bladder by the morbid opening, which he then largely dilated, and easily withdrew the pessary entire. The subsequent treatment and history of the case offer nothing of particular interest, except that a urinary fistula remained after the operation.—*Gazette Med., May,* 1855.

*The Inadvisability of Closing Wound after Operations for Hernia.*—By M. Nelaton.—M. Nelaton is of opinion that every facility ought to be afforded for the escape of any matters which may be formed in the deeper parts of the wound after operations for hernia; for if this provision be not made, the same matter is apt to burrow and excite phlegmonous mischief in the cellular tissue of the neighborhood, particularly in the iliac fossa. M. Nelaton believes that he has lost two patients from this cause, and that many other patients who were supposed to have succumbed from peritonitis, but who presented no signs of peritonitis after death, did, in reality, die from the same cause.—*Nashville Med. and Surg. Journal. Gaz. des Hopitaux.*

*Uterine Displacements.*—The French Academy, by a unanimous vote, have decided against the propriety of using the intra-uterine pessary as being unnecessary and often injurious. After a fair trial of the pessary, we have ourselves long since discarded them, as inconvenient, offensive to delicacy, and positively hurtful. They deserve to be scouted from the profession.—*St. Louis Medical and Surgical Journal.*

*Heavy Damages for Slander of a Physician.*—Dr. O'Neal, of Baltimore, has recently obtained a verdict against a Mr. Jeffries, to the amount of $10,000, for a libel against his professional character. The facts are these: A year ago last July, Jeffries met with a severe railroad accident, by which both legs were fractured, as asserted by Dr. O'Neal, who treated him accordingly. Some six or eight weeks afterwards the patient became dissatisfied, dismissed the Doctor, and contended that the bones had not been fractured, and even went so far as to publish a card in the papers, charging the Doctor with ignorance in not discovering that the limbs were not fractured, and for dishonestly pretending that they were. This was the ground of the libel, and for which the jury, after a long trial, and thorough investigation, awarded the above damages.—*St. Louis Medical and Surgical Journal.*

*Inhalation of the Fumes of Opium in Coryza.*—Dr. Lombard, of Geneva, has found that in those severe cases of coryza which are accompanied by great pain and sense of weight in the frontal sinuses, the inhalation of the fumes of burnt opium affords the patient the most marvelous and speedy relief. The pain ceases as if by enchantment, and the patient passes from a state of misery into one of comfort. Dr. Lombard recommends a few grains of powdered opium to be thrown upon a slip of metal, previously heated in a spirit lamp; and the patient is desired to hold his head over and forcibly inhale the fumes of the drug.—*Gazette Medicale, July*, 1854.

*Sugar and Salt for the Chronic Congestion of the Cervix Uteri.*—F. J. Bumstead, M. D., in the New York Medical Times, recommends the plan of treatment proposed, he believes, by M. Caudmont, of the Ecole Pratique of Paris, for chronic congestion of the cervix uteri. Equal parts of sugar and common table salt, finely pulverized and mixed, are impacted around the vaginal portion of the cervix uteri, through a speculum, and retained for twelve or twenty-four hours. This occasions little pain or inconvenience to the patient, and is more efficacious than leeching or cauterization of these parts.

*Fluid Extracts.*—This convenient and concentrated form for the preservation and administration of medicines is becoming very popular with pharmaceutists—soon we will have fluid extracts of all the vegetable articles of the materia medica. The Journal of Pharmacy announces the fluid extract of ergot among the recent preparations.

